# ß-Globin gene cluster haplotypes in Moroccan sickle cell disease patients: diversity pilot study

**DOI:** 10.4314/ahs.v26i2.7

**Published:** 2026-06

**Authors:** Fatima Zahra Alaoui Ismaili, Touria Derkaoui, Nadia Hamjane, Amina Barakat, Naima Ghailani Nourouti, Mohcine Bennani Mechita

**Affiliations:** 1 Intelligent Automation & BioMed Genomics Laboratory, Faculty of Sciences and Techniques of Tangier, University Abdelmalek Essaâdi, Tangier, Morocco; 2 Higher Institute of Nursing Professions and Health Techniques of Tangier, Ministry of Health,Tangier, Morocco

**Keywords:** Sickle cell Disease, Haplotypes, Hemoglobin F

## Abstract

**Background:**

Sickle cell disease (SCD) is the most common inherited blood disorder worldwide. Although monogenic, it presents substantial clinical heterogeneity influenced by genetic modifiers, including haplotypes and fetal hemoglobin (HbF) levels.

**Objectives:**

This pilot cross-sectional study aimed to characterize, for the first time, the βS gene haplotype distribution among Moroccan patients with sickle cell anemia and evaluate its impact on hematological parameters, particularly HbF levels.

**Methods:**

Eight polymorphic sites within the β-globin gene cluster were analyzed using PCR-RFLP in 334 chromosomes from SCD patients in northern Morocco. Associations between haplotypes and HbF levels were evaluated.

**Results:**

PCR RFLP showed that the Benin haplotype was the most common (61.1%), followed by Bantu (14.1%), Atypical A1 (11.7%), Senegal (10.5%), and Arab-Indian (2.7%). The most frequent genotypes were Ben/Ben (41.3%), Ben/CAR (15%), and Ben/Sen (10.2%). HbF levels varied significantly across haplotypes (p < 0.005), with Senegal and Arab-Indian showing the highest levels and Benin and Bantu the lowest.

**Conclusions:**

This study highlights both the genetic and anthropological diversity of SCD in Morocco, likely reflecting historical African gene flow. Haplotype profiling enhances understanding of genotype-phenotype correlations, offering valuable insights for prognosis and individualized care strategies to improve patients' outcomes.

## Introduction

Sickle cell disease (SCD) is a genetic hemoglobin disorder caused by an amino acid substitution in the β-globin gene cluster [β6(A3) Glu>Val, GAG>GTG], leading to the production of abnormal hemoglobin S (HbS)^1^,^2^. SCD is a significant public health issue, affecting 20–25 million people worldwide, with an estimated 305,800 births annually, 50–60% of which occur in Africa. The disease is associated with high morbidity and mortality rates^3^-^5^.

Although SCD is a monogenic disorder, its clinical presentation is remarkably heterogeneous both within and between populations. Patients may experience a wide spectrum of symptoms, ranging from mild anemia to severe vaso-occlusive crises and life-threatening complications, and increased susceptibility to infections^6^-^8^. The phenotypic variability of SCD is influenced by several genetic modifiers, including β-globin gene cluster haplotypes, linked mutations, co-inheritance of α-thalassemia, levels of fetal hemoglobin (HbF), and the Gγ:Aγ globin chain ratio^9^-^12^. Environmental and socioeconomic factors may also modulate disease expression^13^.

The βS mutation is associated with five major haplotypes, identified by restriction fragment length polymorphism (RFLP) analysis: Senegal, Benin, Bantu/Central African Republic (CAR), Cameroon, and Arab-Indian^14^-^16^. These haplotypes reflect distinct ethnic and geographical origins and are associated with differing HbF levels and clinical outcomes^11^,^17^. However, consistent correlations between haplotype and disease severity remain uncertain.

Due to its geographical position and complex history of migrations, Morocco represents a genetically diverse population. Previous studies have focused on β-thalassemia, but to our knowledge, no prior study has systematically examined the β-globin haplotype background among Moroccan SCD patients. Given the high prevalence of consanguinity and hemoglobinopathies in northern Morocco, particularly in the Larache province, local population studies are essential for understanding the genetic diversity of SCD.

This pilot study aims to address this gap by characterizing the haplotype distribution and investigating their association with HbF levels and hematological indices, especially HbF levels. The findings may offer insights into the genetic history of SCD in the region and contribute to better disease stratification and personalized care.

## Population and methods

A total of 167 patients with confirmed sickle cell disease (HbSS genotype) (M/F ratio = 1.2) were recruited from the provincial hospital in Larache, northern Morocco. Patients with other forms of anemia, those receiving hydroxyurea treatment or recent transfusions, and children under one year of age were excluded to avoid confounding effects on HbF levels.

All participants or their guardians provided informed consent, and the study was approved by the university hospital's ethics committee at the Faculty of Medicine and Pharmacy of Tangier in accordance with the Declaration of Helsinki.

Venous blood samples (4 mL) were collected in EDTA tubes. Hematological indices were measured using an automated hematology analyzer (SYSMEX XT-1800, Kobe, Japan). Hemoglobin variants were identified via capillary electrophoresis (MINICAP, Sebia, Paris, France). Genomic DNA was extracted using the QIAamp® DNA Mini Kit (QIAGEN, Germany), and purity was assessed via NanoDrop spectrophotometry (Thermo Scientific, USA).

Haplotype determination was based on PCR-RFLP analysis of eight polymorphic sites in the β-globin gene cluster, as previously described (18). The PCR products were digested with the appropriate restriction enzymes and visualized on 6% native polyacrylamide gels (29:1). The restriction sites analyzed included: 5′ to ε (HincII), 5′ to Gγ (XmnI), IVS2 of Gγ and Aγ (HindIII), ψβ (HincII), 3′ to ψβ (HincII), IVS2 of β (AvaII), and 3′ to β (HinfI), as listed in Table 1. Haplotypes were reconstructed based on the presence (+) or absence (−) of these sites (Table 3). Atypical haplotypes were those not matching any of the five classical haplotypes.

**Table 1:** The studied regions within the β globin gene cluster; primers' sequence, annealing temperature used in PCR, and amplified product size in bp

The studied region within the β globin gene cluster	Primers' sequence 5′3′		Annealing temperature	Amplified product size in bp
**Hinc II 5′ to ε**	5′ TCTCTGTTTGATGACAAATTC 3′	5′ AGTCATTGGTCAAGGCTGACC 3′	60°C	760
**XmnI 5′ to Gγ**	5′ AACTGTTGCTTTATAGGATTTT3′	5′AGGAGCTTATTGATAACCTCAGAC 3′	54°C	655
**HindIII within IVS II Gγ**	5′ TGCTGCTAATGCTTCATTACAA 3′	5′ AAGTGTGGAGTGTGCACATGA 3′	60°C	780
**HindIII within IVS II Aγ**	5′ TGCTGCTAATGCTTCATTACAA 3′	5′ TAAATGAGGAGCATGCACACAC 3′	63°C	761
**HincII ψβ**	5′GAACAGAAGTTGAGATAGAGA 3′	5′ACTCAGTGGTCTTGTGGGCT 3′	60°C	698
**Hinc II 3**′ **to ψβ**	5′ TCTGCATTTGACTCTGTTAGC 3′	5′ GGACCCTAACTGATATAACTA 3′	54°C	613
**AvaII within IVS II β**	5′ GTCGAGGGTTTGAAGTCCAA 3′	5′ CACTGATGCAATCATTCGTC 3′	54°C	638
**Hinf I 3**′ **to β**	5′ TGGATTCTGCCTAATAAAA 3′	5′GGGCCTATGATAGGGTAAT 3′	54°C	803

**Table 2:** Demographic characteristics and hematological parameters of the study population

Characteristics	Study cohort n =167
**Age group, years**	
1_20	105; 62.6%
20_40	58; 34.8%
>40	4; 2.5%
**Sex**	
Female	78
Male	89
**Hematological data**	
**Hb F (%)**	12.06±5.29
**Hb (g/dl)**	7.86±1.29
**Hb A2 %**	3.72±2.51
**Hb S%**	81.90±5.3
**MCV (fl)**	84.8±9.2
**MCH (pg)**	28.6±4.4

*Values mentioned are mean ± standard deviation. Hb-Hemoglobin, Hb A2-Hemoglobin A2, Hb F-Fetal hemoglobin, Hb S-Sickle Hemoglobin, MCV-Mean corpuscular volume, MCH-Mean corpuscular hemoglobin.*

**Table 3:** Frequency of βS globin gene haplotypes for SCA patients

Restriction enzyme βS Haplotype	Hind II 5′ to ε	XmnI 5′ to Gγ	Hind III Gγ	Hind III Aγ	HincI I ψβ	Hind II 3′ to ψβ	Ava II β	Hinf I 3′ to β	N	Frequency
**Benin**	-	-	-	-	-	+	+	+	**20** **4**	**61.1%**
**Bantu (CAR)**	-	-	+	-	-	-	+	+	**47**	**14.1%**
**Senegal**	-	+	+	-	+	+	+	+	**35**	**10.5%**
**Arab-Indian**	+	+	+	-	+	+	+	-	**9**	**2.7%**
**Atypical 1**	-	-	-	-	-	-	+	+	**39**	**11.7%**
**TOTAL**									**33** **4**	**100%**

*N= Number of chromosomes.*

Statistical analyses were performed using SPSS software, version 20.0 (SPSS Inc., Chicago, IL, USA). Data are expressed as mean ± SD. The Mann-Whitney U test was used to compare HbF levels between haplotypes, and Kruskal-Wallis test was applied for multiple group comparisons. A p-value < 0.05 was considered statistically significant.

## Results

Among the 167 patients with sickle cell disease included in the study, 53% were male (n=89) and 47% were female (n=78), with a sex-ratio of 1.2. The majority of patients (62.6%) were aged between 1 and 20 years (n = 105), 34.8% between 20–40 years (n = 58), and 2.5% were older than 40 (n=4) (Table 1). The demographic and hematological characteristics of the cohort are summarized in Table 2.

A total of 334 chromosomes were analyzed to determine the β-globin haplotype distribution (Table 3). The Benin haplotype was the most common, observed in 61.1% of alleles, followed by Bantu (14.1%), atypical A1 (11.7%), Senegal (10.5%), and Arab-Indian (2.7%). No Cameroon or other atypical haplotypes were detected.

Genotypically, Benin/Benin was the most common (41.3%), followed by Benin/CAR (15%), Benin/Senegal (10.2%) and Benin/A1 (9%). Other genotypes, such as A1/A1 (7.2%), Benin/Arab-Indian (5.4%), CAR/CAR (4.8%), CAR/Senegal (3.6%), and Senegal/Senegal (3.6%), were less frequent (Table 4).

**Table 4:** βS Haplotype Distribution and median values of hematological indices and hemoglobin values by haplotype

Haplotypes	Ben/Ben	Ben/CAR	CAR/CAR	Ben/Sen	Sen/Sen	Ben/Arab-Indian	CAR/Sen	Ben/A1	A1/A1	p global (KW)
N	69	25	15	17	6	9	6	15	12	-
Frequency	41.3%	0,15	4.8%	10.2%	3.6%	5.4%	3.6%	0,09	7.2%	-
HB	8.89±2.85	8.2 ± 1.3	7.9 ± 1.2	10.9±2.41	12.4±2.24	12.03±0.8	9.3± 1.5	8.2 ± 0.82	8.2± 1.9	0.118
HbF%	8.43±3.16	8.3 ±2.2	7.6±3.9	13.6±3.6*	16.4±4.9*	15.4±1.07*	12.6±2.5	8.72±1.51	10.29±2.8	0.020
HbA2%	3.4±1.8	3.35±1.2	3.4±0.4	3.2±0.8	3.7±1.2	3.5±0.4	3.4±0.6	3.12±0.8	3.4±0.5	0.587
HbS%	82.5±5.6	81.3±2.1	82.3±2.5	81.5±3.2	79.4±2.1	80.6±1.3	82.7±2.8	82.90±1.3	82.5±4.7	0.441
MCV	82.77±10.8	78.1±9.1	81.9±14.8	78.9±8.8	77.9±10.7	79.8±1.5	82.5±2.3	81.7±0.8	72.5±9.5	0.305
MCH	27.77±3.4	27.85±3.7	26.93±5.9	26.41±3.4	26.22±4.6	27.15±1.02	27.15±2.6	26.4±1.1	27.65±3.8	0.276

* *Significant difference with Ben/Ben (p<0.005; Mann-whitney U-test)*

**Figure 1:**
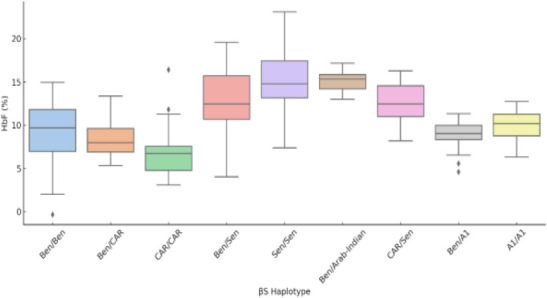
Fetal hemoglobin distribution based on Haplotype

Table 4 summarizes the average hematological values according to haplotype combinations. There was a statistically significant difference in HbF levels across haplotypes (p < 0.001, Kruskal-Wallis test). A boxplot was generated to visualize HbF distribution across haplotypes, further supporting these statistical differences (Fig 1).

Patients carrying the Arab-Indian (Ben/Arab-Indian: 15.4 ± 1.07%) and Senegal (Sen/Sen: 16.4 ± 4.9%) haplotypes exhibited significantly higher HbF levels compared to the Ben/Ben reference group (8.43 ± 3.16%, p < 0.01 and p < 0.005, respectively). Conversely, Bantu-associated haplotypes (CAR/CAR: 7.6 ± 3.9%) were associated with lower HbF levels and more severe hematological profiles. Hemoglobin S levels and other red cell indices, including MCV, MCH, or HbA2 showed no significant variation among haplotypes (all p>0.05), although a trend toward lower hemoglobin levels was noted in Bantu haplotypes.

## Discussion

To our knowledge, this is the first study to investigate the βS haplotype distribution in Morocco, particularly in the Larache province, a region with a high prevalence of hemoglobinopathies. While β-thalassemia haplotypes have been studied in the country^19^,^20^, comprehensive data on the βS mutation has remained scarce.

Our findings show that the Benin haplotype is the most prevalent (61.1%) among Moroccan patients with sickle cell anemia (SCA), followed by the Bantu, atypical A1, Senegal, and Arab-Indian haplotypes. This distribution likely reflects historical gene flow from endemic malaria sub-Saharan Africa into North Africa via trans-Saharan migrations and population admixture^21^,^22^.

Genetic studies have demonstrated that North Africans share significant paternal and maternal lineages with sub-Saharan populations^23^,^24^, supporting a historical continuum. Studies of STR analysis and Y-chromosome polymorphisms support this, showing significant paternal and maternal lineage affinities among North African ethnicities, with over 75% of paternal lineages sharing common ancestry^25^,^26^. Additionally, analysis of mitochondrial DNA control region sequences, tracing maternal lineage, reveals sub-Saharan genetic influences among Maghrebian women^27^,^28^. High rates of consanguinity, culturally common in Morocco, may also contribute to the predominance of homozygous Benin combinations^29^-^31^. The prevalence of Benin haplotype in our cohort aligns with neighboring North African countries, such as Tunisia (60.54%)^32^ and Egypt (80.8%)^33^, as well as in several Middle Eastern countries^11^,^34^,^35^. In contrast, Mauritania shows a predominance of the Senegal haplotype (77.8%), followed by Benin (8.8%), Arab-Indian (5.5%), and Bantu (4.4%)^36^, while in Sudan, the Bantu haplotype predominates alongside a novel Sudan-specific HbS haplotype^37^, highlighting regional diversity. Studies in Spain and France also report a predominance of Benin, mostly among individuals of African origin^38^,^39^. The presence of Senegal and Arab-Indian haplotypes in our Moroccan cohort, although less frequent, suggests additional genetic inputs from West Africa and the Arabian Peninsula, consistent with historical migrations, trans-Saharan trade, and Islamic expansion across the Maghreb to Europe through the Moroccan-Gibraltar corridor^40^.

Interestingly, we identified the atypical A1 [-----++] haplotype at 11.7%, which differs from the Benin haplotype by the 3′ψβ HindII site, and has been previously reported in Tunisia with a high prevalence estimated at 23.49%^41^. This supports the idea that A1 may represent a North African-specific variant. Other atypical haplotypes A [+----++], A2 [-------], and B1 [--+--++] and the Cameroon haplotype were not detected, likely due to geographic and demographic factors.

A key finding of our study is the significant association between βS haplotypes and fetal hemoglobin (HbF) levels. Patients carrying Senegal and Arab-Indian haplotypes had significantly higher HbF levels, a known modifier of disease severity in SCA^12^. HbF inhibits HbS polymerization, reduces red blood cell sickling, and protects against vaso-occlusion and hemolysis^17^. These protective effects were most evident in haplotypes carrying the XmnI (+) site (rs7482144), which is associated with elevated HbF expression^42^,^43^.

Conversely, patients with Benin and Bantu haplotypes exhibited lower HbF levels and more severe hematological profiles, consistent with previous studies that associate these haplotypes with moderate to severe clinical manifestations^9^,^12^. Notably, the Bantu haplotype showed the lowest Hb and MCV values, suggesting greater hemolytic activity. Our findings partially diverge from Okumura et al.^44^, who reported no major hematological differences between Benin and CAR haplotypes, though slight increases in HbF were noted in CAR carriers in our cohort.

Despite the clear association between haplotypes and HbF levels, the variability observed across individuals underscores the multifactorial nature of SCA modulation. HbF expression is shaped not only by β-globin haplotypes but also by major trans-acting regulators such as BCL11A, HBS1L-MYB, KLF1, and other epigenetic factors. The atypical haplotypes identified in our cohort, including Arab-Indian and Senegal, may further contribute to this heterogeneity through distinct cis-regulatory architectures influencing γ-globin activation and HbF persistence^45^-^47^. Although their low frequency limits direct genotype–phenotype analyses, their biological relevance remains substantial. These observations highlight the need for larger, clinically annotated cohorts and comprehensive genomic profiling to clarify the functional and prognostic impact of both common and rare βS haplotypes in the Moroccan population.

Understanding β-globin haplotype diversity in Moroccan Sickle Cell Disease patients is also crucial for practical applications in public health. Knowledge of haplotype distribution can improve risk prediction, guide carrier screening, and support community-focused health education. In regions with high disease prevalence, these insights enable tailored genetic counselling, and informed decision-making, and prenatal screening, ultimately contributing to better disease management and targeted prevention strategies.

## Conclusion

This study is the first to characterize the βS haplotype distribution in a Moroccan population, offering new insights into both the anthropological origins and clinical implications of the βS gene in the region. Mapping haplotype diversity is essential not only for reconstructing the historical migration patterns that shaped the Moroccan gene pool but also for better predicting the clinical severity of sickle cell disease.

The predominance of the severe Benin haplotype observed in our cohort underscores the pressing need for targeted public health interventions. Implementing a nationwide screening and early diagnosis program could significantly improve disease management outcomes and enable the development of more personalized treatment strategies for individuals with sickle cell anemia.

## Limitations

This study has several limitations. First, the relatively small sample size particularly for individuals carrying rare or homozygous haplotypes such as Arab-Indian or Senegal may limit precise estimation of haplotype-specific phenotypic impacts. Second, clinical severity was assessed only indirectly through hematological parameters; no data on vaso-occlusive crises, hospitalizations, or organ damage were available. Third, because the study was conducted in a single region in northern Morocco, the findings may not fully reflect the broader genetic heterogeneity and haplotype distribution across the country, which may affect the generalizability of the results. Finally, although PCR-RFLP is a practical and cost-effective method for β-globin haplotype identification, it provides a less comprehensive assessment of genetic variation compared with sequencing-based approaches, and may overlook rare or novel variants within the globin gene cluster.

To overcome these limitations, future research should adopt broader and more integrative strategies. Large, multi-center cohorts representing different Moroccan regions would allow a more accurate characterization of haplotype diversity and related phenotypes. Incorporating high-resolution molecular techniques, such as next-generation sequencing, would provide a more detailed and reliable assessment of β-globin cluster variation and validate PCR-RFLP-based findings. Future studies should also integrate clinical severity indicators—such as vaso-occlusive crisis frequency, hospitalization history, transfusion burden, and organ involvement—to better clarify the relationship between haplotypes, HbF levels, and disease expression. In addition, expanding the genetic scope to include known modifiers of HbF, such as BCL11A, KLF1, and the HBS1L-MYB intergenic region, as well as examining gene–environment influences and epigenetic interactions would contribute to a more comprehensive understanding of HbF regulation and support the development of more personalized clinical management strategies for patients with SCD.
